# Achieving High-Temperature Measurement Using Thermionic Emission from a W–La_2_O_3_ Cathode in Low-Pressure Argon Glow Discharge

**DOI:** 10.3390/ma19112230

**Published:** 2026-05-25

**Authors:** Wei Liang, Xinyu Zhang, Jun Wang, Fanlei Wu, Kai Zhang, Qun Cao, Minggang Tang

**Affiliations:** 1 Taihu Laboratory of Deepsea Technological Science, Wuxi 214000, China; zhangxy@cssrc.com.cn (X.Z.); wangjun@cssrc.com.cn (J.W.); wufl@cssrc.com.cn (F.W.); kaizhang@cssrc.com.cn (K.Z.); caoqun@cssrc.com.cn (Q.C.); tangmg@cssrc.com.cn (M.T.); 2 China Ship Scientific Research Center, Wuxi 214082, China

**Keywords:** high-temperature measurement, metallic composites, thermal properties, thermionic emission characteristics, thermionic emission-driven glow discharge

## Abstract

This study investigates the feasibility of obtaining high-temperature (2000–2200 °C) measurements using thermionic emission from a W–La_2_O_3_ cathode in a low-pressure argon glow discharge environment. Compared to a vacuum environment, the cathode emission characteristics and temperature variation patterns in a plasma environment exhibit significant differences. These differences arise primarily from the competitive interplay between the thermionic emission cooling (TEC) effect and the ion bombardment heating (IBH) effect. Among the discharge parameters (temperature, applied bias voltage, and background pressure), the applied bias voltage is the key factor influencing this competitive interplay. Consequently, the cathode surface temperature exhibits three distinct regions as a function of bias voltage: the TEC-dominated region (10–20 V), the transition region (20–40 V), where TEC and IBH are nearly in equilibrium, and the IBH-dominated region (40–60 V). The results indicate that by adjusting the discharge parameters to place thermionic emission in the transition region, the TEC and IBH effects can be mutually offset. Under these conditions, the cathode temperature can be unambiguously determined from the measured emission current using the modified Schottky equation. This approach simplifies the functional relationship between emission current and temperature (*J*–*T*), thereby enabling high-temperature measurements to be obtained.

## 1. Introduction

Temperature monitoring plays a crucial role in many fields, including medicine, aerospace, and hydrogen energy [1,2,3]. Measurement devices covering a temperature range from −273.13 °C to 2726.85 °C have been developed [4,5]. The accurate assessment of the high-temperature resistance of materials is essential in scenarios where structural temperatures rise sharply, such as in the aerodynamic heating of hypersonic vehicles or combustion and explosions caused by hydrogen leaks [6,7]. Moreover, the precise, real-time temperature monitoring of critical heat-shielding, fire-resistant, and blast-resistant components is a key prerequisite for designing safety margins, evaluating the severity of disasters, and initiating emergency responses [8,9]. Optical pyrometers are currently the most widely used instruments for high-temperature measurement. After calibration against blackbody radiation and correction for deviations, their accuracy can reach within 1% [10]. A drawback of optical pyrometers, however, is that they require a direct line of sight and are subject to limitations on the monitoring distance [11,12]. As a result, researchers have begun to explore the use of thermionic electron emission from materials in a vacuum environment for temperature measurement [13,14]. This phenomenon is known as the Richardson effect, in which the emission current maintains a functional relationship with temperature (denoted as the *J–T* relationship). Studies have confirmed that this method can cover a wide temperature range and holds potential for practical applications.

Recent work on thermionic emission vacuum diodes for temperature measurement has shown that a novel vacuum diode operating in the barrier-lowering regime can serve as a temperature sensor [15]. Unlike semiconductor materials, thermionic emission diodes can operate stably in air without degradation [16]. In addition, electrons are neither scattered by the crystal lattice nor trapped by defects during transport [17]. Thermionic electrons are therefore generally regarded as more suitable than solid-state electrons for harsh environments or applications requiring fast-response monitoring [18]. To overcome the limitations imposed by the space charge effect, an external electric field is normally required to assist thermionic emission. In a vacuum environment, the gap between the electron-emitting surface and the collector must typically be kept in the submicron to sub-10 μm range [19,20]. This places stringent demands on fabrication precision and cost [21,22], and also limits the development of large-area devices. By contrast, in a low-pressure glow discharge plasma, positive ions can partially compensate for the electron space charge [23], allowing the electrode gap to be relaxed to the millimeter scale [24]. Increasing the electrode separation from the nanometer/micrometer level to the millimeter level significantly eases the fabrication tolerance, reduces the cost, and facilitates the construction of larger devices suitable for structural-scale monitoring. At the same time, the low-pressure inert gas environment eliminates the need for ultra-high vacuum. Introducing a plasma environment, however, brings about a new problem: the cathode surface is simultaneously subjected to thermionic emission cooling (TEC) and ion bombardment heating (IBH) [25,26,27], and the resulting cathode temperature is governed by the competitive interplay between these two effects. This competitive interplay has been the subject of extensive theoretical and numerical studies in atmospheric-pressure arcs and microdischarges. Benilov et al. [28] systematically compared different approaches to modeling the near-cathode space-charge sheath and demonstrated that the macroscopic characteristics of the plasma–cathode interaction are predominantly controlled by local processes—namely, ion heating, electron emission cooling, and radiation—rather than by the remote arc column. This finding suggests that the cathode thermal state can be manipulated through local discharge parameters. Building on this theoretical framework, Baeva et al. [29] developed a unified non-equilibrium model of a tungsten-inert-gas microarc in atmospheric-pressure argon. Their work explicitly resolved the individual contributions of ion bombardment, thermionic emission, secondary emission, and plasma electron heating to the total heat flux at the cathode surface, and showed that, in the arc regime, the current is carried almost entirely by thermionic electrons. Saifutdinov [24] performed a comprehensive numerical study of various DC discharge modes in atmospheric-pressure argon and further demonstrated that, due to the competitive interplay between TEC and IBH, the cathode surface temperature exhibits a non-monotonic dependence on the discharge current, with distinct regimes dominated by one effect or the other. A recent topical review by Baeva and Uhrlandt [30] summarized the state of the art in microarc characterization and modeling, noting that virtually all reported studies have been carried out at atmospheric pressure and have aimed primarily at understanding the discharge physics. The unified modeling approach has also been extended to transient discharges. For instance, Santos et al. [31] simulated the ignition of an AC arc on initially cold electrodes in atmospheric-pressure argon and tracked the sequence of current-transfer mechanisms: displacement current, ion current, and, finally, thermionic emission as the cathode heats up. Although that study focused on a non-steady ignition phase, it illustrates the versatility of the unified methodology and reinforces the picture that thermionic emission becomes the dominant electron source once a sufficiently high temperature is reached.

The works cited above have significantly deepened the understanding of the TEC–IBH competitive interplay in glow discharge plasmas and its influence on cathode temperature and discharge modes. However, these studies have been motivated by discharge physics or material processing, without involving temperature measurement applications. In the field of thermometry, existing methods based on thermionic emission have been developed exclusively for vacuum environments [13,14]. To the best of our knowledge, no previous work has directly employed thermionic emission in a glow discharge plasma as a means of temperature measurement. Furthermore, the effect of gas composition on the emission characteristics of a hot cathode cannot be ignored. For W–La_2_O_3_ cathodes, it has been shown that La_2_O_3_ is highly sensitive to both oxidizing and reducing environments at high temperatures. In a reducing atmosphere, carbon can reduce La_2_O_3_ to an active La monolayer that is essential for maintaining a low work function [32]. In an oxidizing atmosphere, H_2_O can dissociatively adsorb on the La_2_O_3_ surface to form hydroxyl groups [33], and CO_2_ can poison La_2_O_3_ by forming stable surface carbonates [34]. These findings indicate that changes in the gas environment may significantly alter the surface chemistry and emission properties of La_2_O_3_. For this reason, high-purity argon was chosen as the working gas in this study so as to exclude chemical interference from the gas environment and to focus on the competitive interplay between TEC and IBH. The thermometry approach developed on this basis can be extended to other gas environments in future work.

In summary, prior studies have greatly advanced the understanding of the TEC–IBH competitive interplay, but this physical mechanism has not yet been transformed into a practical engineering measurement tool. The present work starts from this observation and aims to explore, in a low-pressure argon glow discharge, whether the TEC–IBH competition can be balanced by adjusting the discharge parameters, thereby reducing the relationship between emission current density and temperature to a single-valued form that can be described by the modified Schottky equation. To test this idea, a high-temperature-resistant W–La_2_O_3_ hot cathode was prepared, and both experimental and numerical investigations were carried out. In the experiments, the emission current and the cathode surface temperature were measured in a low-pressure argon glow discharge. In the numerical simulations, a fluid model accounting for the TEC–IBH competitive interplay was established to systematically examine the influence of the discharge parameters on the emission characteristics and the temperature response of the cathode. By adjusting parameters such as the bias voltage, both the experiments and the simulations identified a transition region where TEC and IBH are nearly balanced, and it was confirmed that, in this region, the cathode temperature can be determined from the measured emission current using the modified Schottky equation, thereby validating the feasibility of thermionic-emission-based temperature measurement in a low-pressure glow discharge.

## 2. Materials and Methods

To prepare the W–La_2_O_3_ composite powder, W powder (≥99.9%, 200 nm; Macklin, Shanghai, China, Cat. No. 768160) and 8 wt% La_2_O_3_ powder (≥99.99%, 50 nm; Macklin, Shanghai, China, Cat. No. L812321) were mixed using a high-energy ball milling process in anhydrous ethanol (AR, ≥99.7%, Sinopharm Chemical Reagent Co., Ltd., Shanghai, China). After drying the mixed powder in an argon atmosphere, it was sintered using spark plasma sintering (SPS) technology at 1600 °C for 10 min under a uniaxial pressure of 40 MPa. During the sintering process, the residual pressure in the vacuum chamber was maintained at approximately 10 Pa. Upon completion of sintering, the resulting W–La_2_O_3_ composite material was processed into a disc-shaped cathode measuring ∅10 mm × 1 mm.

**Figure 1:**
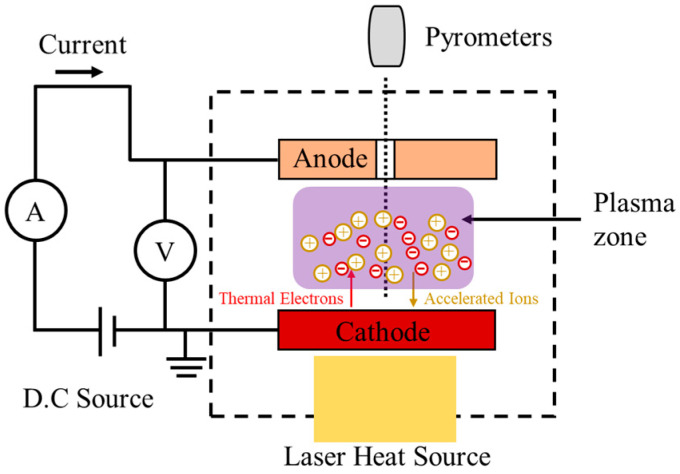
Schematic diagram of the testing system.

As shown in Figure 1, the test system used in this experiment is capable of creating a low-pressure argon environment (10–100 Pa) or a vacuum environment (with a background pressure maintained at approximately 1 × 10^−4^ Pa). The cathode is heated using a continuous-wave fiber laser with a wavelength of 980 nm. The cathode surface temperature was measured using a two-color pyrometer (DIAS DSRF 11 N, DIAS Infrared GmbH, Dresden, Germany; temperature range 799–2500 °C, repeatability 0.1% of reading, resolution 0.1 °C). The instrument determines temperature from the ratio of radiation intensities at two adjacent near-infrared wavelengths, and was factory-calibrated against a blackbody radiation standard traceable to PTB. Plasma radiation in this spectral region is dominated by discrete spectral lines [35], with an intensity at least seven orders of magnitude lower than the cathode thermal radiation at the experimental temperatures, and therefore does not measurably affect the readings.

The emission current was measured using a multimeter (Fluke-8845A, Fluke Corporation, Everett, WA, USA; accuracy 0.045%). The distance between the cathode and anode was set to 1 mm. A DC power supply was used to apply a positive potential (<100 V) to the anode relative to the cathode. Prior to all experiments and characterization measurements, the W–La_2_O_3_ cathode was preheated to approximately 2300 °C and held at that temperature for 5 min to ensure stability and consistency during the subsequent testing process. This pretreatment not only allowed the cathode to reach a stable thermionic emission state but also removed adsorbed moisture and oxygen-containing impurities from the cathode surface through high-temperature desorption, thereby stabilizing the surface condition prior to measurement.

The microstructure of the samples was characterized using a scanning electron microscope (SEM, TESCAN MIRA LMS, TESCAN, Brno, Czech Republic) and an X-ray diffractometer (XRD, Rigaku Ultima IV, Rigaku, Tokyo, Japan). The work function of the samples was measured using an ultraviolet photoelectron spectrometer (UPS, Thermo Fisher, ESCALAB 250Xi, Waltham, MA, USA). The density, thermal conductivity, and emissivity of the samples were measured using a density meter (ETNAL ET-120H, Beijing Etnal Electronic Technology Co., Ltd., Beijing, China; measured value: 9.5 g/cm^3^), a laser thermal conductivity meter (Nettleland LFA-427, Netzsch, Selb, Germany; measured value: 15.59 W/m·°C), and an infrared radiometer (InfraRed FTIR-16, InfraRed Associates, Inc., Stuart, FL, USA; measured value: 0.41), respectively.

To facilitate the interpretation of the experimental data, the measurement uncertainties associated with the key quantities are evaluated below. A brief evaluation of the measurement uncertainties is given here. In the present experimental system, the cathode surface temperature and the emission current density are not independent measurands; rather, they are two strongly coupled aspects of the same physical process, linked through the thermionic emission equation and the cathode energy balance. Consequently, the uncertainties of the two quantities share the same set of fundamental perturbation sources, and the evaluation can be carried out within a unified framework.

The shared perturbation sources consist primarily of three contributions. The first is temperature fluctuation, which originates from the repeatability of the pyrometer (±2 K). This fluctuation contributes ±2 K to the temperature uncertainty itself, while simultaneously introducing a relative uncertainty of approximately 1.7% in the current density through the exponential sensitivity of the thermionic emission equation. The second is pressure fluctuation (±1 Pa). In classical cold-cathode glow discharges, the scaling relationship between current density and pressure typically follows $J\propto p^{n}$. In the present work, however, the discharge is a hot-cathode-assisted non-self-sustained discharge. Electrons originate primarily from thermionic emission, and the direct influence of pressure on the current density is far weaker than in the classical case (n=2) [36]. Therefore, adopting n=2 represents a highly conservative estimate. Under this very conservative assumption, the relative uncertainty introduced by pressure fluctuations does not exceed 4.0%. The third contribution is bias voltage fluctuation (±0.5 V). The bias voltage affects the current density indirectly through the Schottky effect by modifying the surface electric field. The sensitivity coefficient relating the current density to the surface electric field in the Schottky term is very small (in the order of 10^−3^ in relative terms), and the resulting contribution to the current density uncertainty is far below 1%. The bias voltage fluctuation can also influence the cathode temperature through the ion bombardment heating effect. Under the typical conditions of this study, the temperature variation caused by a ±0.5 V fluctuation is estimated to be less than 1 K, which is within the pyrometer repeatability. This contribution is therefore already accounted for in the temperature uncertainty and requires no separate treatment. Combining the uncertainty contributions of the above perturbation sources to both the emission current and the temperature yields a total relative standard uncertainty of no more than approximately 4.4%. As a reference for evaluating the significance of instrumental contributions, the error bars shown for the experimentally measured emission current and temperature data in this paper represent the standard deviation of at least three independent repeat measurements. The systematic uncertainty estimated above is substantially smaller than these standard deviations. A further discussion of the relationship between the two is presented in Section 4.

## 3. Numerical Methods

Under specific discharge conditions, a direct-current glow discharge driven by thermal electrons between parallel plate electrodes can form a stable plasma. By adjusting the discharge parameters, it is possible to control the thermionic emission flux as well as the mass and energy fluxes of charged particles transported to the cathode surface. However, because both the TEC effect and the IBH effect on the cathode surface must be considered simultaneously, this problem is difficult to solve directly using analytical or semi-analytical methods. Therefore, computational fluid dynamics (CFD) methods were used in this study to numerically simulate the thermionic emission process on the cathode surface under glow discharge conditions, along with the resulting temperature response.

### 3.1. DC Glow Discharge Model

**Figure 2:**
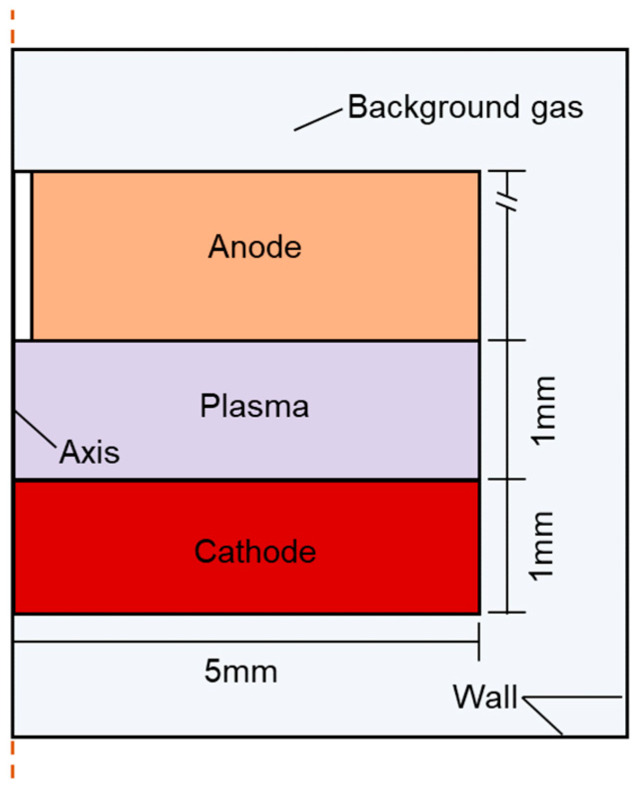
Plasma modeling with parallel plate electrodes.

A model of the DC glow discharge region between the experimental parallel-plate electrodes was established, as shown in Figure 2. The figure illustrates the division of the simulation domain and the corresponding names. A DC power supply drives the discharge by applying a voltage across the parallel-plate gap, thereby establishing an axial electric field between the electrodes. High-purity argon (Ar, ≥99.999%, Harbin Liming Gas Co., Ltd., Harbin, China) is used as the working gas. Given the axial symmetry of the discharge region, a two-dimensional axisymmetric model is adopted. The computational domain is defined as a longitudinal cross-section encompassing the inter-electrode discharge region.

This discharge plasma model primarily involves four types of particles: free electrons, atoms, metastable atoms, and ions (e^−^, Ar, Ar^+^, Ar*), as shown in Table 1. It also includes seven plasma chemical reactions [37] and two surface reactions [38,39] in which these particles participate.

### 3.2. Control Equation

This study employed a two-dimensional axisymmetric fluid dynamics model to simulate cathode thermionic emission and surface temperature response in a direct-current glow discharge plasma environment between parallel-plate electrodes. The primary governing equations involved are as follows.

Drift-diffusion transport equations for electrons and heavy particles [44]:

(1) $$\frac{\partial n_{e}}{\partial t}+\nabla \cdot \Gamma_{e}+\left(u_{g}\cdot \nabla \right)n_{e}=R_{e}$$

(2) $$\frac{\partial n_{\varepsilon }}{\partial t}+\nabla \cdot \Gamma_{\varepsilon }+\Gamma_{\varepsilon }\cdot E+(\begin{array}{c} u_{g}\cdot \nabla \end{array})n_{\varepsilon }=R_{\varepsilon }$$

(3) $$\rho_{g}\frac{\partial w_{i}}{\partial t}+\rho_{g}\left(u_{g}\cdot \nabla \right)w_{i}+\nabla \cdot \Gamma_{i}=R_{i}$$

In the above equations, $n_{e}$ and $n_{\varepsilon }$ are the electron density and electron energy density, respectively. $\Gamma_{e}$ and $\Gamma_{\varepsilon }$ are the electron flux and electron energy flux, respectively. $R_{e}$ is the electron collision source term, representing the creation and annihilation of electrons. $R_{\varepsilon }$ is the change in electron energy during the reaction, $u_{g}$ is the velocity of the flowing neutral gas, and E is the electric field strength. Furthermore, $\rho_{g}$ is the density of the working gas, $w_{i}$ is the mass fraction of the i-th species, $R_{i}$ is the heavy-particle collision term, and $\Gamma_{i}$ is the flux of the i-th species. Note that $R_{e}$, $R_{\varepsilon }$, and $R_{i}$ are all derived from the plasma chemical kinetic reactions in Table 1.

The Poisson equation is used to calculate the electric field, as given in Equation (4):

(4) $$-\nabla^{2}\varphi =\frac{e}{\varepsilon_{0}}\left(\sum_{i=1}^{N}Z_{i}n_{i}-n_{e}\right)$$

where φ, $\varepsilon_{0}$, $n_{i}$ and e are the plasma potential, vacuum permittivity, ion number density, and elementary charge, respectively.

The Navier–Stokes equations for an inert gas and the convection–diffusion heat transfer equation are as follows:

(5) $$\frac{\partial \rho_{g}}{\partial t}+\nabla \cdot \left(\rho_{g}u_{g}\right)=0$$

(6) $$\rho_{g}\frac{\partial u_{g}}{\partial t}+\rho_{g}\left(u_{g}\cdot \nabla \right)=\nabla \cdot \left[-pI+\mu_{d}\left(\nabla u_{g}+{\left(\nabla u_{g}\right)}^{T}\right)-\frac{2}{3}\mu_{d}\left(\nabla \cdot u_{g}\right)I\right]$$

(7) $$\rho_{g}C_{p,g}\frac{\partial T_{g}}{\partial t}+\rho_{g}C_{p,g}u_{g}\cdot \nabla T_{g}+\nabla \left(-k_{g}\nabla T_{g}\right)=Q_{hs}+Q_{J,ion}$$

In the above equations, p is the pressure of the mixture, $\mu_{d}$ is the dynamic viscosity, and I is the unit tensor. $T_{g}$ is the neutral gas temperature, $C_{p,g}$ is the specific heat of the gas, and $k_{g}$ is the gas thermal conductivity. $Q_{hs}$ is the energy exchange term between electrons and heavy particles, and $Q_{J,ion}$ is the Joule heating term for ions. These source terms are expressed as follows:

(8) $$Q_{e}=\frac{3}{2}k_{B}(\frac{2m_{e}}{m_{i}})(T_{e}-T_{g})v_{eh}$$

(9) $$Q_{J,ion}=J_{\mathrm{ion}}\cdot E$$

where $k_{B}$ is the Boltzmann constant, $m_{e}$ is the electron mass, $m_{i}$ is the heavy–particle mass, $T_{e}$ is the electron temperature, and $J_{\mathrm{ion}}$ is the ion current density. The term $v_{eh}$ is the total electron–heavy-particle collision frequency, which is determined from the plasma chemical reaction.

Assuming the cathode material is homogeneous and isotropic, and in the absence of internal heat sources, the equation of thermal conduction equilibrium for the cathode is as follows:

(10) $$\rho_{c}C_{p,c}\frac{\partial T_{c}}{\partial t}+\nabla \left(-k_{c}\nabla T_{c}\right)=0$$

where $\rho_{c}$ is the density of the cathode material, $C_{p,c}$ is the specific heat capacity of the cathode material, $k_{c}$ is the thermal conductivity of the cathode material, and $T_{c}$ is the cathode temperature. The specific values of these parameters are provided in the experimental section.

### 3.3. Initial Conditions and Boundary Conditions

The initial electron density is set to 1 × 10^6^ m^−3^, the initial average electron energy to 4 eV, and the initial potential to 0 V. During gas discharge, reduction reactions occur on the electrode surfaces. Therefore, the boundary conditions for the electron flux, electron energy flux, and heavy-particle flux at the cathode and anode surfaces are as follows:

(11) $$\Gamma_{e}\cdot n=\frac{1}{2}n_{e}\sqrt{\frac{8k_{B}T_{e}}{\pi m_{e}}}-\left[\sum_{i}\gamma_{i}\left(\Gamma_{i}\cdot n\right)+\Gamma_{t}\cdot n\right]$$

(12) $$\Gamma_{\varepsilon }\cdot n=\frac{5}{6}n_{\varepsilon }\sqrt{\frac{8k_{B}T_{e}}{\pi m_{e}}}-\left[\sum_{i}\gamma_{i}\varepsilon_{i}(\Gamma_{i}\cdot n)+\varepsilon_{t}\Gamma_{t}\cdot n\right]$$

(13) $$\Gamma_{i}\cdot n=M_{i}R_{s,i}+w_{i}\rho \mu_{i,m}Z_{i}(E\cdot n)$$

In the above equations, n is the normal vector of the boundary, $\varepsilon_{i}$ is the average energy of secondary electrons, $\varepsilon_{t}$ is the average energy of thermal electrons, and $\gamma_{i}$ is the secondary emission coefficient, taken as a typical empirical value of 0.25 for such cathode materials. $M_{i}$ is the mole mass of the heavy particles, $R_{s,i}$ is the wall reaction rate, and $\mu_{i,m}$ is the mobility of the heavy particles.

The thermionic emission flux is derived from the thermionic emission current density as follows:

(14) $$\Gamma_{t}=J_{t}/e$$

where the emission current density is given by $n\cdot J_{t}=J_{e}$, and $J_{e}$ is the Schottky-enhanced thermionic emission current density [45]:

(15) $$J_{e}=J_{RD}\exp\left(\frac{e}{k_{B}T_{c}}\sqrt{-\frac{eE_{sur}}{4\pi \varepsilon_{0}}}\right)$$

(16) $$J_{RD}=A_{RD}T_{c}^{2}\exp\left(-\frac{ew_{f}}{k_{B}T_{c}}\right)$$

In this equation, $J_{RD}$ is the Richardson thermionic emission current density, $A_{RD}$ is the Richardson constant (120 A·°C^−2^·m^−2^), $w_{f}$ is the surface work function of the material, and $E_{sur}$ is the Schottky effect electric field. Here, $E_{sur}$ is defined as the local electric field normal to the cathode surface, obtained self-consistently at the cathode boundary from Poisson’s equation (Equation (4)). It is not a preset constant but results from the iterative coupling with the space-charge distribution.

To facilitate calculations in this model, the cathode potential is defined as 0 V. A positive anode potential corresponds to a negative bias applied to the cathode relative to the anode. Thus, the total discharge current ($I_{a-tol}$) at the anode boundary is expressed as follows:

(17) $$I_{a-tol}=\int_{\Omega }\left(n\cdot J_{i}+n\cdot J_{e}+n\cdot J_{d}\right)dS$$

where $J_{i}$ is the ionic displacement current density and, $J_{d}$ is the electron displacement current density.

For the gas flow equation, a no-slip boundary condition is applied to the interface between the electrodes and the fluid computational domain. The remaining boundaries are set as fixed-pressure boundaries. In the gas heating equation, the anode wall is maintained at a fixed temperature of 26.85 °C. For the cathode wall, a heat flux boundary condition is imposed [46]:

(18) $$-eq_{c}\cdot n=-n\cdot J_{e}\left(w_{f}+2k_{B}T_{c}\right)+n\cdot J_{i}\left(E\left(\lambda_{exc}\right)+V_{i}-w_{f}\right)-\varepsilon \sigma T_{c}^{4}+\left(-k_{g}\nabla T_{g}\right)$$

The first term on the right-hand side is the heat flux ($Q_{TEC}$) corresponding to the TEC effect, including secondary electron emission and thermionic emission. The second term is the ion bombardment heat flux ($Q_{IBH})$, where $\lambda_{exc}$ is the ion charge exchange distance, and $V_{i}$ is the first ionization energy of neutral particles. The third term is the surface thermal radiation heat flux. The final term represents thermal convection of the neutral gas, with an initial gas temperature of 26.85 °C.

## 4. Results and Discussion

**Figure 3:**
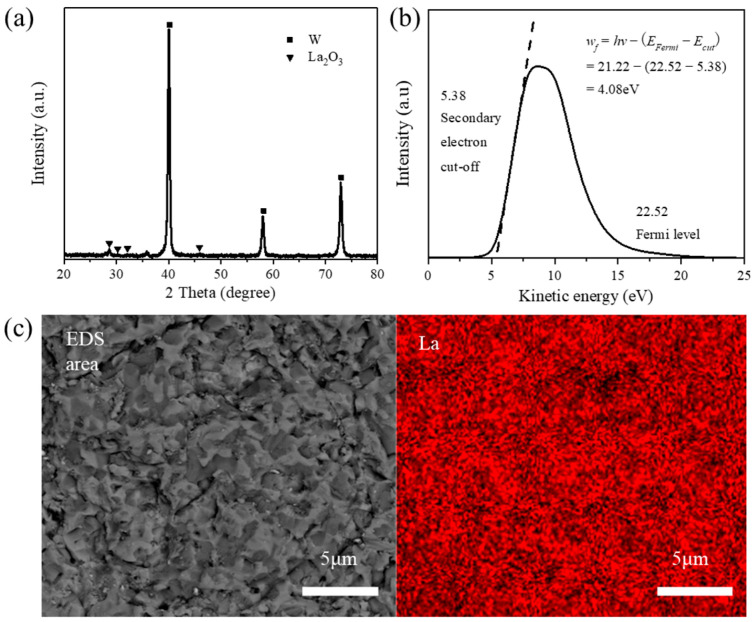
XRD pattern (**a**), UPS spectrum (**b**), and SEM micrograph and EDS map (**c**) of W–8%La_2_O_3_ cathode.

The X-ray diffraction (XRD) pattern shows that the nanocomposite cathode consists of two phases: W and La_2_O_3_ (Figure 3a). Within the detection limits of XRD, no reaction products between W and La_2_O_3_ or other impurity phases were observed. The ultraviolet photoelectron spectroscopy (UPS) results indicate that the work function of the W–8%La_2_O_3_ cathode is 4.08 eV (Figure 3b). Scanning electron microscopy (SEM) images of the cathode reveal that the lanthanum oxide nanoparticles form a finely dispersed island-like structure with tungsten, with sizes ranging from several micrometers to 10 micrometers (Figure 3c). Energy-dispersive X-ray spectroscopy (EDX) mapping reveals that La is uniformly distributed across the surface of the W–8%La_2_O_3_ cathode. Because the La_2_O_3_ dispersed in the matrix has a lower work function, it serves as the preferred electron emission center. These microstructural characteristics indicate that the composite cathode can be approximated as an ideal emitter, with electron emission capability tending to be consistent across all surface regions.

**Figure 4:**
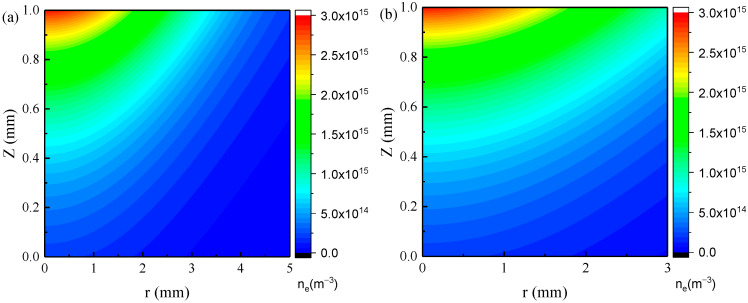
Spatial distribution of electron number density between cathode and anode at 2150 °C, 50 Pa, and 30 V: (**a**) r ≤ 5 mm; (**b**) r ≤ 3 mm.

Figure 4 shows the spatial distribution of electrons during a stable discharge state within the gap between parallel plate electrodes. This result was obtained through numerical simulation under the conditions of 2150 °C, 50 Pa, and 30 V. As shown in Figure 4a,b, the discharge region is primarily concentrated within the cross-sectional range of r ≤ 3 mm, where the radial variation in electron number density is relatively small. Specifically, within the range of r ≤ 2 mm, the variation is even smaller, so that the radial electron distribution can be regarded as essentially uniform. Beyond this central region, the electron density drops significantly, indicating that the emission current is highly localized within the discharge-covered area. Consequently, the contribution from the peripheral region to the total current is negligible, and the thermionic emission characteristics are dominated by the central uniform emission zone.

**Figure 5:**
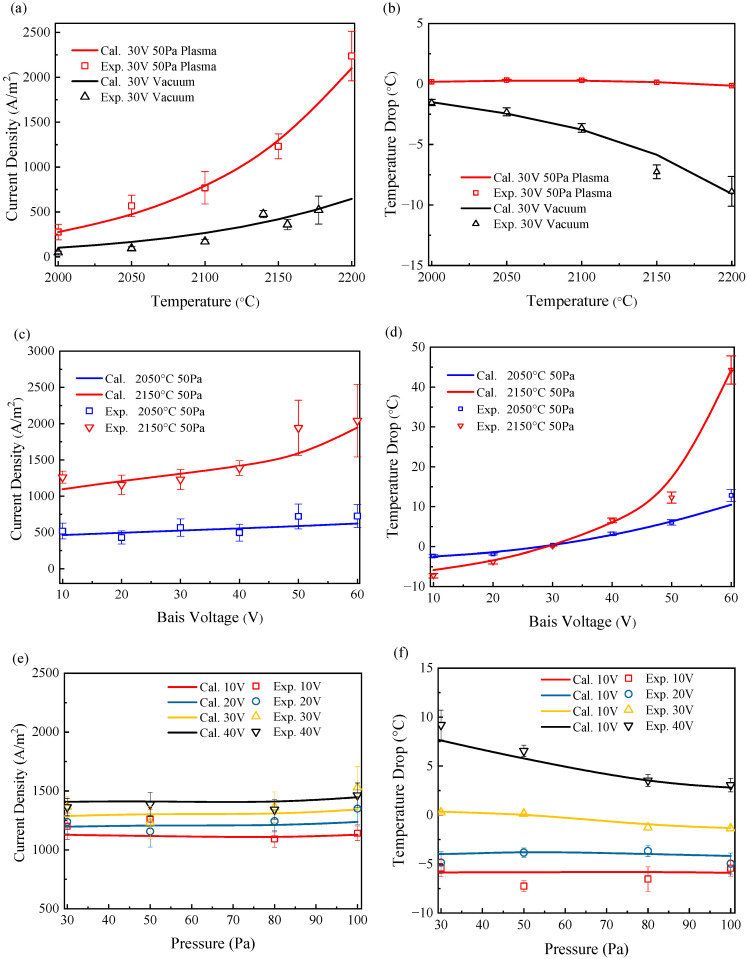
Emission characteristics (*J–T*, *J–V*, and *J–P*) and temperature drop (*T_D_*–*T*, *T_D_*–*V*, and *T_D_–P*) of W–La_2_O_3_ cathode obtained from experimental testing and numerical calculation: (**a**) *J–T* and (**b**) *T_D_–T* in vacuum and in plasma (50 Pa, 30 V); (**c**) *J–V* and (**d**) *T_D_–V* at 2050 °C and 2150 °C (both at 50 Pa); (**e**) *J–P* and (**f**) *T_D_–P* at 2150 °C with bias voltages of 10, 20, 30, and 40 V. In all panels, error bars represent the standard deviation of at least three independent repeated measurements.

Figure 5 shows the experimental and numerical calculation results for the thermionic emission current and temperature dependence of a W–La_2_O_3_ cathode under different discharge conditions. As noted in Section 2, the systematic uncertainty is substantially smaller than the experimental standard deviations. This indicates that the observed data scatter originates primarily from intrinsic physical fluctuations of the thermionic emission–plasma coupled system, rather than from instrumental limitations. First, the differences between plasma and vacuum environments are compared. As shown in Figure 5a, the emission current density in the argon discharge environment is significantly higher than that in the vacuum environment, and the difference between the two increases with rising temperature. Estimations using Equation (15) indicate that the Schottky electric field on the cathode surface in the plasma environment is approximately two orders of magnitude higher than that in the vacuum environment. This suggests that the presence of plasma further reduces the surface potential barrier compared to the vacuum environment, thereby enhancing the surface’s electron emission capability. However, the TEC effect associated with the significantly increased thermionic emission current did not result in a more pronounced cooling effect compared to the vacuum environment; instead, a slight increase in surface temperature was observed, as shown in Figure 5b. It is evident that the presence of a plasma environment not only alters the thermionic emission characteristics of the cathode but also modifies the pattern of cathode surface temperature variation. Consequently, the Richardson equation, which is applicable to vacuum environments, cannot accurately describe thermionic emission in the discharge environment. In contrast, calculation results that simultaneously account for the thermionic emission effect (TEC) and the internal resistance heating effect (IBH) are in better agreement with experimental results and can effectively describe the underlying physical processes.

Figure 5c,d present the variations in steady-state emission current density and surface temperature with increasing bias voltage at 2050 °C and 2150 °C, both under a pressure of 50 Pa. As shown in Figure 5c, the cathode emission current increases monotonically with the bias voltage, and this trend becomes more pronounced at higher temperatures. In contrast to the monotonic increase in current, the cathode surface temperature exhibits a distinctly non-monotonic dependence on the bias voltage. As shown in Figure 5d, a significant decrease in cathode temperature is observed only when the applied bias voltage is within the range of 10–30 V. When the applied bias voltage exceeds 30 V, the cathode surface temperature no longer decreases but instead gradually increases. Although the applied bias further lowers the cathode’s surface potential, thereby enhancing the Schottky effect, it also causes Ar^+^ ions in the plasma to be accelerated by the electric field and bombard the cathode surface, causing its temperature to rise. Therefore, when the cathode potential is lower than the plasma potential (i.e., under negative bias conditions), surface heating caused by ion bombardment is inevitable. Further comparison of the results under different temperature and applied bias conditions reveals that the cathode temperature primarily affects the thermionic emission current, whereas the applied bias affects both TEC and IBH. It is preliminarily concluded that a critical threshold voltage exists, which governs the relative strengths of the TEC and IBH effects, thereby determining whether the cathode temperature rises or drops.

In addition to bias voltage and cathode temperature, background pressure is a key parameter governing the discharge characteristics. It is necessary to further investigate its influence on both thermionic emission and cathode temperature. Given that the preceding analysis indicates that the cathode temperature is weakly affected by ion bombardment heating, the cathode temperature is fixed at 2150 °C. The subsequent analysis focuses on the evolution of emission current density and surface temperature with increasing background pressure under various bias voltages. As shown in Figure 5e,f, when the bias voltage is below 30 V, changes in cathode temperature are primarily attributed to the thermionic emission cooling (TEC) effect. Under these conditions, the effect of pressure variations on the emission current density and cathode temperature is far weaker than under high bias voltages and is not significant within the parameter range investigated here. When the bias voltage exceeds 30 V, the ion bombardment heating (IBH) effect becomes dominant. However, its intensity gradually decreases with increasing background pressure, and the attenuation of the IBH effect increases with increasing bias voltage. This phenomenon can be attributed to the dominant physical mechanism at low bias voltages. At these low bias voltages (<30 V), the discharge operates in the thermionic emission cooling (TEC)-dominated regime. The cathode surface temperature and thermionic emission flux are determined primarily by the baseline thermal state of the cathode and the Schottky effect, neither of which is sensitive to pressure variations. In this regime, the ion bombardment heating (IBH) effect contributes little to the overall energy balance at the cathode surface. Changes in the plasma collision frequency and ion flux induced by pressure variations are therefore insufficient to produce a significant effect on the cathode temperature or emission current within the parameter range investigated. As the bias voltage increases into the IBH-dominated regime (>40 V), ion bombardment becomes a major factor influencing the cathode temperature. The intensity of ion bombardment depends directly on ion generation and transport, and both processes are closely coupled to the gas pressure. The influence of pressure becomes evident at high bias voltages.

**Figure 6:**
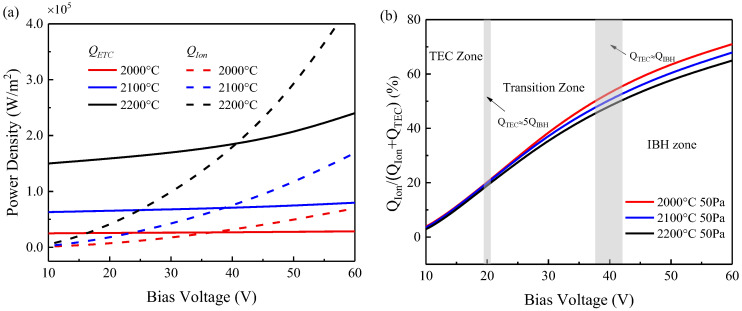
Calculated results of (**a**) $Q_{TEC}$ and $Q_{IBH}$, and (**b**) $Q_{IBH}/\left({Q_{TEC}+Q}_{IBH}\right)$ under different applied bias voltages at cathode temperatures of 2000, 2100, and 2200 °C.

Based on the analysis of the above results, the TEC and IBH effects in a plasma environment compete with each other in influencing the cathode surface temperature. In the following discussion, the patterns of their effects on surface temperature changes and their relative contributions are further examined using the calculated values of TEC power density ($Q_{TEC}$) and IBH power density ($Q_{IBH}$). As shown in Figure 6a, within the bias voltage range of approximately 10 V, $Q_{TEC}$ is significantly greater than $Q_{IBH}$, and TEC dominates at this stage. As the applied bias voltage increases to the 30–40 V range, the values of $Q_{TEC}$ and $Q_{IBH}$ become comparable. When the bias voltage increases further, the influence of IBH on the surface temperature exceeds that of TEC, and IBH becomes the dominant factor. Therefore, the variation in the cathode surface temperature with applied bias in a plasma environment can be divided into three regions: the TEC region dominated by space-charge limitations (10–20 V), the transition region (20–40 V), and the bombardment heating region (40–60 V), as shown in Figure 6b. When the applied bias drives thermionic emission into different regions, the corresponding heat transfer mechanisms govern the energy flux at the cathode surface. In the TEC region, both the thermionic emission current density and the heat dissipation power density increase with the applied bias, leading to a corresponding decrease in cathode temperature. In the transition region, the energy released by ion acceleration and impact increases significantly as the electric field strengthens, resulting in $Q_{TEC}\approx Q_{IBH}$. Consequently, the cathode temperature remains approximately constant. When the applied bias voltage increases further and enters the bombardment heating region, $Q_{TEC}$ becomes lower than $Q_{IBH}$. At this stage, the cathode surface temperature is dominated by IBH and rises significantly with increasing bias voltage.

Combining the simulation and experimental results, this study elucidates the influence of bias voltage on the thermionic emission characteristics and surface temperature of the cathode. The results indicate that bias voltage is the dominant factor governing the variation in cathode surface temperature, and that the surface temperature exhibits a characteristic zonation pattern as a function of bias voltage. In the transition region (30–40 V), the $Q_{TEC}$ and $Q_{IBH}$ values are close. At this point, the bias voltage can be set in the transition region (for example, 30 V at 2000–2200 °C and 50 Pa) so that the TEC and IBH effects cancel each other out, and the cathode temperature remains approximately constant. Based on this, the experimentally validated Schottky-enhanced thermionic emission equation (Equation (15)) can be used to calculate the real-time surface temperature from the emission current. This method effectively simplifies the *J−T* relationship and provides a feasible approach for high-temperature measurement using thermionic emission from W–La_2_O_3_ cathodes in low-pressure argon glow discharge.

The stability of the cathode material under the present experimental conditions warrants a brief discussion here. La_2_O_3_ possesses high thermodynamic stability. Its melting point is approximately 2315 °C [47], far above the operating temperature range of this study (2000–2200 °C). Within this temperature range, evaporation loss is negligible. Moreover, the testing sequence from 2200 °C down to 2000 °C further reduces the risk of material degradation at the highest temperatures. It should be noted that the present work focuses on the competition mechanism between TEC and IBH, and a systematic evaluation of the long-term thermal cycling stability of the cathode has not yet been undertaken. Dedicated cycling tests combined with detailed microstructural characterization will be carried out before this measurement approach is deployed in practical applications.

## 5. Conclusions and Outlook

In summary, this paper describes the fabrication of a W–La_2_O_3_ cathode with uniform thermionic emission characteristics using the spark plasma sintering method. The feasibility of high-temperature measurements based on thermionic emission in a low-pressure argon glow discharge environment was verified through both experiments and numerical simulations. Both experimental and computational results indicate that, under identical conditions, the emission current density in the plasma environment is significantly higher than that in a vacuum environment. This implies that, to achieve the same emission level, the plasma environment imposes less stringent parameter requirements on thermionic emission, and the fabrication and processing conditions for the corresponding devices are less demanding. Compared to vacuum conditions, the emission characteristics and temperature response of the cathode in a plasma atmosphere exhibit significant differences. These differences arise primarily from the competition between two effects: thermionic emission cooling (TEC) and ion bombardment heating (IBH). Among discharge parameters such as temperature, bias voltage, and gas pressure, the applied bias voltage plays a decisive role in this competitive interplay. As a result, the cathode surface temperature exhibits three distinct regions as the bias voltage varies: the TEC-dominated region (10–20 V), the transition region (20–40 V, where TEC and IBH are nearly in equilibrium), and the IBH-dominated region (40–60 V). By adjusting the discharge parameters such that thermionic emission operates within the transition region, the TEC and IBH effects mutually offset, allowing the cathode temperature to remain approximately constant. The surface temperature can then be inferred from the emission current using the experimentally validated Schottky-enhanced thermionic emission equation. This approach simplifies the *J−T* relationship and enables high-temperature measurement based on thermionic emission from a W–La_2_O_3_ cathode in low-pressure argon glow discharge. The method established and the principles identified in this study provide a theoretical and experimental foundation for the development of low-cost, easy-to-manufacture sensors designed for complex environments such as high-temperature conditions.

The present model reasonably captures the integral *J−T* and *J−V* characteristics and reveals the key competitive mechanism between TEC and IBH. However, the current validation relies primarily on two macroscopic quantities—total emission current and cathode surface temperature. The spatial distributions of electron density, electric potential, and electron temperature have not yet been compared with independent experimental data in a spatially resolved manner. Although these predicted distributions are internally self-consistent, direct experimental confirmation is still needed. Future work will focus on exploring non-invasive diagnostic techniques suitable for the conditions of this study, aiming at a systematic validation of the model’s spatial distributions.

## Figures and Tables

**Table 1:** Important reaction processes in argon discharge.

No.	Reaction Equation	Reaction Type
R_1_	e^−^ + Ar → e^−^ + Ar	Elastic collisions [37]
R_2_	e^−^ + Ar → e^−^ + Ar*	Excitation of the ground state [40]
R_3_	e^−^ + Ar* → e^−^ + Ar	De-excitation [40]
R_4_	e^−^ + Ar → 2 e^−^ + Ar^+^	Ionization of the ground state [40]
R_5_	e^−^ + Ar* → 2 e^−^ + Ar^+^	Stepwise ionization [40]
R_6_	Ar* + Ar* → Ar^+^ + Ar + e^−^	Penning ionization [41,42,43]
R_7_	Ar* + Ar → 2Ar	Metastable decay [41,42,43]
R_8_	Ar* → Ar	Wall reactions [38,39]
R_9_	Ar^+^ → Ar	Wall reactions [38,39]

## Data Availability

Due to its proprietary nature, supporting data cannot be made openly available. The data are available from the corresponding author upon reasonable request.
